# The mechanism of wen jing tang in the treatment of endometriosis: Insights from network pharmacology and experimental validation

**DOI:** 10.1016/j.heliyon.2024.e39292

**Published:** 2024-10-17

**Authors:** Xufang Hu, Xiaoya Guo, Dongxu Wei, Jingyi Yue, Jian Zhang, Bing Wang

**Affiliations:** School of Basic Medicine, Heilongjiang University of Chinese Medicine, Harbin, 150040, China

**Keywords:** Network pharmacology, Endometriosis, Wen jing Tang, HIF1 signaling pathway, Inflammation

## Abstract

**Background:**

Endometriosis (EM) is a hormone-dependent condition marked by progressively severe secondary dysmenorrhea, significantly impacting patients' quality of life and overall health. Wen Jing Tang (WJT), a traditional Chinese medicinal formulation derived from the *Synopsis of the Golden Chamber*, has proven to be an effective therapeutic agent for EM. However, the precise molecular mechanisms underlying its efficacy remain unclear.

**Objective:**

This study aims to elucidate the underlying mechanisms of WJT in the treatment of EM by integrating network pharmacology analysis with experimental validation.

**Methods:**

The chemical constituents and target sites of WJT were obtained from the TCMSP database, while EM-related target genes were sourced from OMIM, TTD, GeneCards, and the DrugBank databases. A "herbs-components-targets" network was constructed using Cytoscape 3.9.1. The intersecting target genes of WJT and EM were then uploaded to the STRING database for protein-protein interaction (PPI) analysis. Subsequently, the common target genes were subjected to GO and KEGG enrichment analysis via the DAVID database. Molecular docking were employed to analyze the binding affinities between the top five core components and their respective targets. Additionally, ELISA were used to quantify the serum levels of IL-6, IL-1β, E2, and P in EM model rats. The expression levels of TNF-α, HIF1A, STAT3, and EGFR mRNA and proteins in ectopic endometrial tissue were assessed using q-PCR and Western blotting.

**Results:**

A total of 250 chemical components and 553 targets were identified in WJT, while 3491 EM-related targets were screened from multiple databases. Among these, 187 common targets between WJT and EM were found, with quercetin, kaempferol, and beta-sitosterol emerging as the core chemical components, and AKT1, IL6, TNF, and IL1B identified as the key targets. These core components demonstrated strong binding affinities to the targets. GO and KEGG enrichment analyses revealed that the shared targets were primarily involved in the HIF1 signaling pathway. Furthermore, compared to the control group, the EM model rats exhibited an increased ectopic endometrial area, disordered glandular and stromal cells, and notable inflammatory infiltration. Serum levels of IL-6, IL-1β, E2, and P were significantly elevated (*P* < 0.01), and the expression of TNF-α, HIF1A, STAT3, and EGFR in the ectopic endometrium was markedly increased (*P* < 0.01). Following WJT intervention, the ectopic endometrial area in model rats was reduced, the morphology and structure of the endometrial cells showed improvement, and serum levels of IL-6, IL-1β, E2, and P were significantly decreased (*P* < 0.05). WJT also inhibited the expression of HIF1 pathway-related proteins TNF-α, HIF1A, STAT3, and EGFR (*P* < 0.05).

**Conclusion:**

The mechanism by which WJT prevents and treats EM may involve the reduction of inflammation through the inhibition of the HIF1 signaling pathway.

## 1 Introduction

Endometriosis (EM) is a chronic inflammatory condition primarily characterized by pain, mass formation, and infertility. The hallmark of EM is the presence of endometrial-like tissue outside the uterus, with common implant sites being the pelvic cavity, ovaries, rectum, and bladder. These ectopic tissues contribute to a chronic inflammatory response, predominantly driven by estrogen [1,2]. EM is one of the most prevalent gynecological disorders in premenopausal women, with studies indicating that approximately 2%–22 % of women of reproductive age may be affected [3]. The prevalence rises to 40%–60 % among women suffering from dysmenorrhea [4], and 25%–50 % of infertile women are found to have EM [5]. Despite its prevalence, the exact pathogenesis and mechanisms underlying EM remain unclear, though inflammation, oxidative stress, and genetic predispositions are believed to play critical roles [6]. Sampson's theory of "retrograde menstruation" is currently the most widely accepted explanation for the development of EM [7]. During menstruation, endometrial fragments, cells, and protein-rich fluids are thought to flow back through the fallopian tubes into the pelvic cavity. Many of these shed cells, including immune cells such as neutrophils, monocytes, and macrophages, activate inflammatory mediators like IL-6, IL-8, and TNF-α [8]. This inflammatory cascade is believed to be the primary driver of EM lesion formation and the source of the associated pain symptoms [9]. Additionally, immune cell infiltration can lead to lipid peroxidation and a subsequent reduction in the activity of antioxidant enzymes, such as catalase (CAT), superoxide dismutase (SOD), and glutathione peroxidase (GSH-Px) [10]. Simultaneously, oxidative stress markers, including reactive oxygen species (ROS) and 8-iso-PGF2α, tend to increase [11]. Clinically, the primary treatment options for EM include hormonal therapies, non-steroidal anti-inflammatory drugs (NSAIDs), and aromatase inhibitors. However, while these medications help alleviate symptoms, they do not fully reverse the disease process and are often accompanied by undesirable side effects [12].

In traditional Chinese medicine, EM is attributed to a deficiency of yang qi in the kidneys, which impairs blood circulation and leads to chronic pain. Wenjing Tang (WJT), a classical TCM formula originating from the ancient medical text *Synopsis of the Golden Chamber*, is composed of 12 herbs (Table 1). This formulation is traditionally believed to replenish yang qi and enhance blood circulation, suggesting its potential therapeutic benefits for treating EM [13]. Pharmacological research has shown that WJT possesses multiple bioactive effects, including the enhancement of ovarian microvascular circulation, regulation of hormone levels, prevention of spontaneous abortion, and treatment of infertility [[14], [15], [16]]. However, the precise mechanisms by which WJT exerts its therapeutic effects on EM remain to be elucidated.

**Table 1:** Herbal ingredients and dosage of WJT.

Botanical plant names	Chinese name	Amount (g)
*Angelica sinensis* (Oliv.) Diels	Dang Gui	10
*Paeonia lactiflora* Pall.	Bai Shao	10
*Cinnamomi Ramulus*	Gui Zhi	10
*Tetradium ruticarpum* (A. Juss.) T.G.Hartley	Wu Zhu Yu	15
*Chuanxiong Rhizoma*	Chuan Xiong	15
*Zingiber Officinale Roscoe*	Sheng Jiang	15
*Pinellia ternata* (Thunb.) Ten. ex Breitenb.	Ban Xia	15
*Cortex Moutan*	Mu Dan Pi	15
*Ophiopogon japonicus* (L. f.) Ker Gawl.	Mai Dong	15
*Panax ginseng* C. A. Mey.	Ren Shen	15
*Asini Corii Colla*	E Jiao	15
*Glycyrrhiza uralensis* Fisch.	Gan Cao	15

**Fig. 1:**
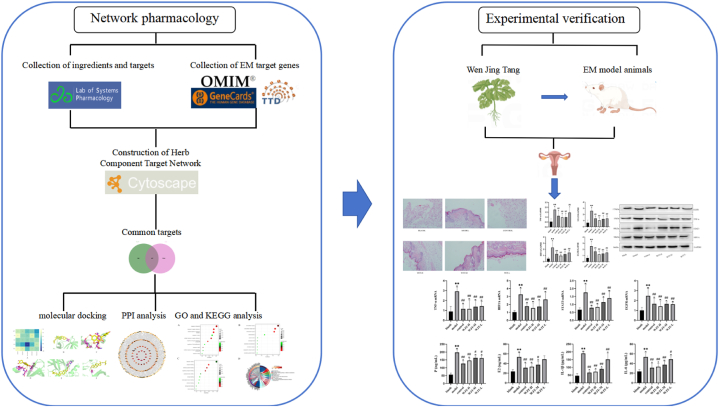
Flowchart of the study Workflow.

Network pharmacology is an emerging approach in systems biology that facilitates the connection between the chemical components of botanical drugs and disease targets. By leveraging various bioinformatics databases, it enables the analysis of gene function to elucidate the molecular mechanisms through which natural products exert therapeutic effects [17]. In this study, we employed network pharmacology to investigate the underlying mechanisms of WJT in the treatment of EM and validated our findings through in vivo experiments (Fig. 1). Our goal is to provide valuable insights and references for the application of traditional Chinese medicine (TCM) in the treatment of EM.

## 2 Materials and methods

### 2.1 Animals

A total of 60 healthy female Sprague-Dawley (SD) rats, with an average weight of 225.31 ± 8.78 g, were supplied by Liaoning Changsheng Biotechnology Co., Ltd. (License No: SCXK (Liao) 2021-0001). The rats were housed in a controlled laboratory environment at a temperature of 25–27 °C and a relative humidity of 40%–70 % for one week. During the acclimatization period, estradiol valerate (0.2 mg/kg) was administered once daily to synchronize the estrous cycle of the rats. This study was approved by the Experimental Ethics Committee of Heilongjiang University of Traditional Chinese Medicine (Approval No: SYXK2020-004).

### 2.2 Drugs and reagents

The composition and respective amounts of the herbs that constitute WJT are detailed in Table 1. All herbs were sourced from the pharmacy of the First Affiliated Hospital of Heilongjiang University of Chinese Medicine and were authenticated by Professor Sun Huifeng. The preparation process involved soaking the medicinal materials in 10 times their volume of water for 30 min, followed by a 30-min decoction to extract the first portion of the liquid. Subsequently, 8 times the volume of water was added for a second 30-min decoction, after which the liquid was strained again. The two extracts were then combined and concentrated to a final concentration of 2 g/mL, and the resulting decoction was stored at 4 °C for later use [18].

The following materials and reagents were used in the study: Estradiol valerate and ethinylestradiol cyproterone tablets (Bayer Weimar GmbH und Co. KG, Lot No. 649A, KT0CTAP); hematoxylin-eosin solution (Beijing Reagan Biotechnology Co., Ltd); ELISA kits for estradiol (E2) (Cat: SEKR-0107), progesterone (P) (Cat: P9060) (Beijing Solebao Technology Co., Ltd); ELISA kits for interleukin-6 (IL-6) (Cat: ab259341), interleukin-1β (IL-1β) (Cat: ab234437), and tumor necrosis factor α (TNF-α) (Cat: ab307164) (Abcom Trading Co., Ltd); SDS (Sigma-Aldrich Company); PageRuler™ Prestained Protein Ladder (Thermo Fisher Scientific Inc.); antibodies against HIF1A (Cat: ab179483), STAT3 (Cat: ab32500), EGFR (Cat: ab316155), TNF-α (Cat: ab307164), and GAPDH (Cat: ab8245) (Abcom Trading Co., Ltd); and goat anti-rabbit secondary antibody (Cat: bs0295G) (Wuhan Sanying Biotechnology Co., Ltd).

### 2.3 Screening of chemical composition and disease targets

The Traditional Chinese Medicine Systems Pharmacology (TCMSP) database (http://tcmspw.com/tcmsp.php) was utilized to identify the chemical constituents of WJT. The screening criteria were set as oral bioavailability (OB) ≥30 % and drug-likeness (DL) ≥ 0.18 to ensure the selection of active compounds. The chemical composition data obtained were uploaded to the PharmMapper platform (http://www.lilab-ecust.cn/pharmmapper/) and subsequently converted into standard gene names using the UniProt database (https://www.uniprot.org/).For endometriosis-related target genes, a search was conducted in the OMIM database (http://www.omim.org), TTD database (http://bidd.nus.edu.sg/), GeneCards database (https://www.genecards.org), and DrugBank (https://www.drugbank.ca). These databases provided the relevant EM-associated target genes. The intersection of WJT-related and EM-related targets was identified using Venny 2.1.0 to determine the common targets for further analysis.

### 2.4 Analysis of protein-protein interaction

Upload the intersection targets to the STRING database (htts://string-db.org/) with the biological species option set to "*Homo sapiens*", the minimum interaction threshold set to "Highest confidence>0.9″ and the other parameters set to their default values, and download the tsv file. The core and hub target genes sets were screened using CytoHubba and MCODE plug-ins, and a visual protein-protein interaction (PPI) network was constructed.

### 2.5 Construction of the “herbs-components-targets” network and enrichment analysis

DAVID database (https://david.ncifcrf.gov/tools.jsp) was used in the intersection of target gene ontology (GO) analysis and the enrichment of KEGG, with *P* < 0.05 for limiting conditions, cell components (CC), biological process (BP), Molecular function (MF) and enrichment pathway were visualized by R language.

### 2.6 Molecular docking

To perform molecular docking, the structural data files (SDF) of the active ingredients are first obtained from the PubChem database. These SDF files are then converted into the Mol2 format for compatibility with further analysis. Next, the 3D structures of the key targets are retrieved and downloaded from the RCSB PDB database (https://www.rcsb.org). Using AutoDock Tools 15.6 software, the protein receptors undergo preprocessing steps such as dehydration and hydrogenation, and are then exported as PDBQT files. Similarly, the small molecule ligands are structurally modified and also exported in PDBQT format. After preparing the receptor and ligand files, docking parameters and docking ranges are defined. The docking of active ingredients with the key targets is then performed using AutoDock Vina, allowing for the assessment of the binding affinities and interactions between the molecules.

### 2.7 Modeling and administration

**Fig. 2:**
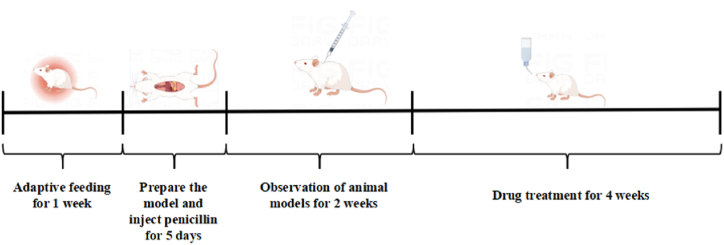
Modelling and administration point.

Sixty Sprague-Dawley (SD) rats were randomly assigned to six groups: blank, model, control, WJT high-dose (WJT-H), WJT medium-dose (WJT-M), and WJT low-dose (WJT-L), with 10 rats in each group. Following the administration of estradiol valerate, all rats were anesthetized via intraperitoneal injection of 5 % pentobarbital sodium. An incision was made in the abdominal skin, and a 0.5 cm^2^ section of endometrial tissue, excised from the upper 1 cm of the rat's uterus, was autografted onto the peritoneum and sutured in layers. In contrast, the blank group underwent laparotomy, where only the adipose tissue surrounding the uterus was removed without endometrial transplantation. Upon successful establishment of the animal model, rats received intramuscular injections of penicillin once daily for 5 days to prevent postoperative infection. Two weeks post-surgery, the model was considered successfully established if the contralaterally transplanted endometrium developed into transparent vesicles containing fluid [19]. For treatment, rats in the control group received 0.18 mg/kg of ethinylestradiol cyproterone solution via intragastric administration. The WJT-H, WJT-M, and WJT-L groups received Wenjing Tang decoctions at doses of 22.5 g/kg, 11.25 g/kg, and 5.63 g/kg, respectively, also via intragastric administration. The model and blank groups were administered an equivalent volume of distilled water. All treatments were administered once daily for 4 weeks (Fig. 2).

### 2.8 HE and IHC staining

The ectopic endometrial tissues were imfixed in formalin solution, and then dehydrated with gradient alcohol, treated with xylene transparency, and embedded with paraffin. The sections were dewaxed and hydrated, then stained with hematoxylin-eosin, dehydrated with alcohol step by step and fully transparent with xylene, then sealed with gum, observed under a microscope and photographed. Endometrial sections were immersed in PBS for thermal repair treatment using EDTA, subsequently immersed in 3 % hydrogen peroxide for 3min, dripping with HIF1A (1:800) and STAT3 (1:800) primary antibodies, and incubated for 16h at 4 °C. Wash with PBS three times and add secondary antibody, incubated for 20 min in 37 °C, adding DAB, subsequently immersed in hematoxylin counterdye for 2 min, followed by gradient alcohol dehydration, xylene penetration and plate sealing.

### 2.9 ELISA

Blood samples from the rats were obtained via the abdominal aorta using a blood sampling technique. Following centrifugation, the supernatant was carefully collected. The absorbance values were then measured using ELISA, in strict accordance with the manufacturer's protocol for the ELISA kit. The expression levels of estradiol (E2), progesterone (P), interleukin-6 (IL-6), and interleukin-1β (IL-1β) were subsequently quantified.

### 2.10 Q-PCR detection

A 100 mg sample of ectopic endometrial tissue was weighed and homogenized in 1 mL of TRIzol reagent using a tissue grinder. The homogenate was centrifuged at 12,000 r·min⁻^1^ for 10 min, after which the supernatant was collected and mixed with chloroform. The RNA-containing aqueous phase was then combined with isopropanol and centrifuged again for 10 min to isolate the total RNA. The extracted RNA was subsequently reverse-transcribed into complementary DNA (cDNA) using a reverse transcription kit (ChamQ Universal SYBR qPCR Master Mix). Quantitative PCR (qPCR) was performed under the following cycling conditions: predenaturation 95 °C for 30s, 95 °C for 10s, 60 °C for 20s, cycling 40 times; 95 °C for 15s, 60 °C for 60s, 95 °C for 15s. The relative expression levels of mRNA for each target gene were calculated using the 2^-ΔΔCt^ method. The primers used in the study were synthesized by Shanghai IBSBIO Biotechnology Co., Ltd., with sequences provided in the supplementary tables.

### 2.11 Western blot detection

A 200 mg sample of ectopic endometrial tissue was combined with RIPA lysis buffer and homogenized thoroughly at 4 °C. The homogenate was then centrifuged, and the supernatant was collected. Protein concentration was determined using a BCA protein assay kit. SDS-PAGE was performed using a 10 % separating gel and a 5 % stacking gel, with electrophoresis conducted at 80 V for 30 min followed by 120 V for 50 min. Proteins were transferred onto a PVDF membrane, which was blocked with 5 % skim milk at room temperature for 1.5 h. The membrane was then incubated overnight at 4 °C with the following diluted primary antibodies: TNF-α (1:1000), HIF1A (1:1000), STAT3 (1:1000), EGFR (1:1000), and GAPDH (1:10,000). After washing with TBST, a diluted secondary antibody was applied. Protein bands were visualized using ECL supersensitive chemiluminescent detection reagent, and the gray values of the bands were quantified for statistical analysis using ImageJ software.

### 2.12 Statistical analysis

The obtained data were processed and analyzed using SPSS 25.0 and GraphPad Prism 8.0 software. The measurement data were presented as mean ± standard deviation (SD). Statistical analysis was performed using one-way analysis of variance (ANOVA) when the data met parametric assumptions. For non-parametric data, the Kruskal-Wallis H test was employed. A P-value of less than 0.05 was considered statistically significant.

## 3 Results

### 3.1 WJT components and EM target screening results

**Fig. 3:**
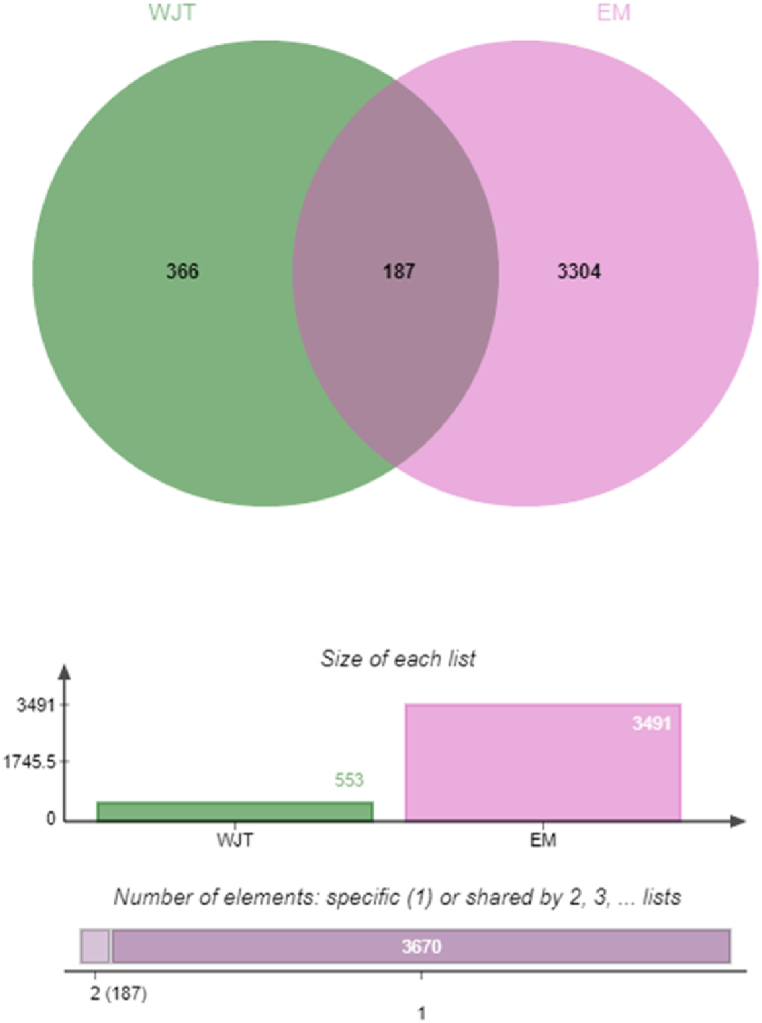
**Venn diagram illustrating the intersection of targets between WJT and EM.** The green represents the 553 drug targets identified in Wenjing Tang (WJT), while the pink represents the 3491 disease-related targets associated with endometriosis (EM). The overlap between these two datasets indicates 187 common targets, highlighting potential therapeutic targets for WJT in the treatment of EM.

A total of 250 chemical constituents were identified in Wenjing Tang (WJT). The herbs Gui Zhi, Wu Zhu Yu, Mai Dong, Dang Gui, Bai Shao, Chuan Xiong, Ren Shen, E Jiao, Mu Dan Pi, Sheng Jiang, Ban Xia, and Gan Cao were found to contain 7, 30, 39, 2, 13, 7, 22, 9, 11, 5, 13, and 92 compounds, respectively. After eliminating duplicate entries, a total of 553 unique targets were obtained. From the databases OMIM, TTD, GeneCards, and DrugBank, 3491 EM-related target genes were retrieved. Following the intersection of WJT targets with EM-associated targets, 187 common targets were identified (Fig. 3).

### 3.2 Results of the construction of the herbs - components - targets network

**Fig. 4:**
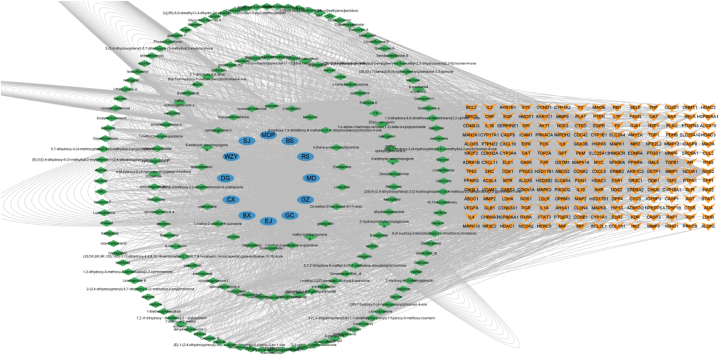
**Network of "Herbs - Components - Targets".** The blue modules represent the constituent herbs of WJT, where MDP refers to Mu Dan Pi, BS to Bai Shao, RS to Ren Shen, MD to Mai Dong, GZ to Gui Zhi, GC to Gan Cao, EJ to E Jiao, BX to Ban Xia, CX to Chuan Xiong, DG to Dang Gui, WZY to Wu Zhu Yu, and SJ to Sheng Jiang. The green modules represent the chemical components of these herbs, and the yellow module indicates the intersection targets shared by WJT and EM.

The chemical components of WJT and the intersecting targets were imported into Cytoscape 3.9.1 to construct a "herbs-component-target" network (Fig. 4). As depicted in the figure, a total of 182 chemical components from the 12 medicinal herbs were ultimately identified. Among these, the core chemical components were quercetin, kaempferol, beta-sitosterol, isorhamnetin, stigmasterol, glycine, rutaecarpine, sitosterol, medicarpin, and glabrene. These 10 compounds are suggested to be the key bioactive components of WJT in the treatment of EM (Table 2).

**Table 2:** Core chemical composition.

Chemical composition	Degree	Betweenness centrality	Closeness centrality
quercetin	276	0.009	1
kaempferol	148	0.004	1
beta-sitosterol	106	0.006	1
isorhamnetin	46	0.0006	1
stigmasterol	39	0.0005	1
glycine	24	0.0005	1
rutaecarpine	22	0.0002	1
sitosterol	21	0.0004	1
medicarpin	20	0.00001	1
glabrene	20	0.0000001	1

### 3.3 PPI network analysis results

**Fig. 5:**
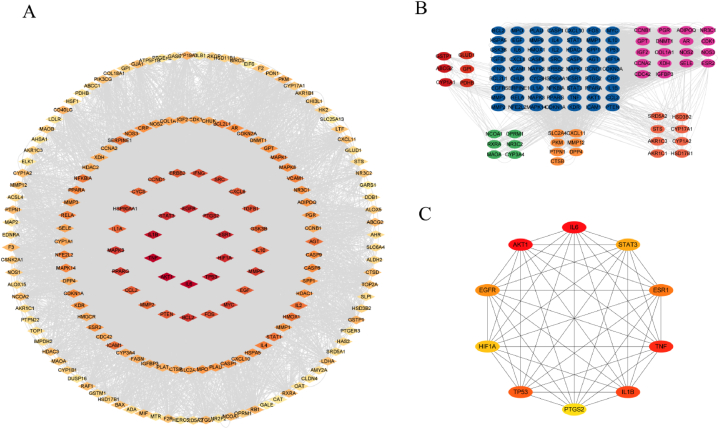
**PPI analysis.** (A) Protein-Protein Interaction (PPI) network of intersection targets: In this network, node color represents significance, with redder nodes indicating higher degree values and yellower nodes indicating lower values. The top 10 targets identified are AKT1, IL6, TNF, IL1B, TP53, ESR1, EGFR, STAT3, HIF1A, and PTGS2. (B) Intersection target gene module map: Seven distinct gene modules were identified within the intersection target set, with the blue module being the largest, consisting of 68 target genes. (C) Core target interaction map: This map displays the interactions among the top 10 core targets. AKT1 was found to have the highest degree value, signifying its prominent interaction with other targets in the network.

The intersection targets were uploaded to the STRING platform, resulting in a network consisting of 185 nodes and 4160 edges. This network was then imported into Cytoscape 3.9.1 for visual analysis (Fig. 5A). Using the MCODE plug-in, seven distinct gene modules were identified, with the blue module being the largest, containing 63 target genes and 718 edges (Fig. 5B). The top 10 core target genes were selected using the CytoHubba plug-in, namely AKT1, IL6, TNF, IL1B, TP53, ESR1, EGFR, STAT3, HIF1A, and PTGS2 (Table 3, Fig. 5C). These core genes may play crucial roles in the mechanism of WJT in treating EM.

**Table 3:** Core targets.

Targets	Degree	Betweenness centrality	Closeness centrality
AKT1	130	3.23	0.78
IL6	130	2.96	0.78
TNF	129	3.06	0.77
IL1B	122	2.71	0.75
TP53	119	2.41	0.74
ESR1	114	3.18	0.73
EGFR	112	2.30	0.72
STAT3	111	1.21	0.72
HIF1A	110	1.66	0.72
PTGS2	109	2.08	0.71

### 3.4 Analysis results of GO and KEGG enrichment

**Fig. 6:**
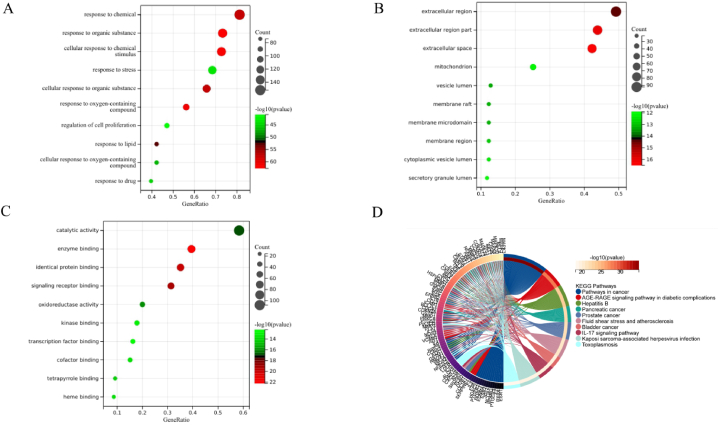
**Enrichment analysis** (A) GO enrichment analysis BP spectrum; (B) GO enrichment analysis of CC spectrum; (C) GO enrichment analysis of MF spectrum; (D) KEGG enrichment analysis.

**Fig. 7:**
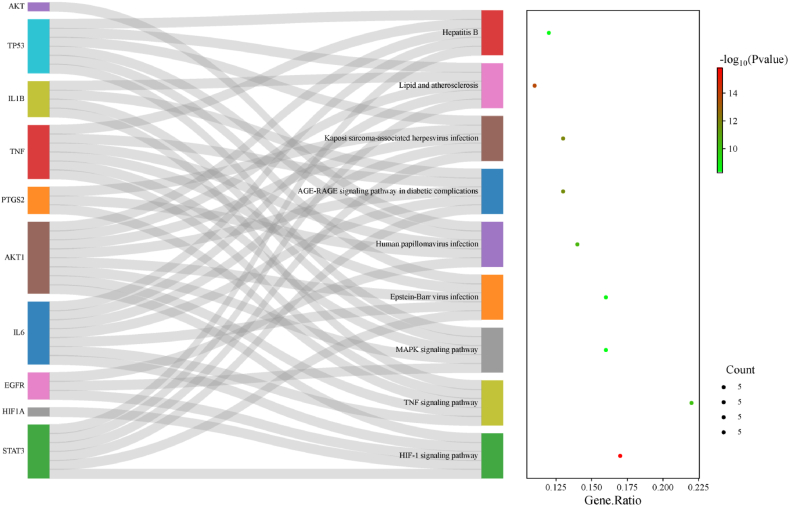
**Sankey Diagram of Core targets-pathway.** The left side of Sankey diagram is the core target, and the right side is the pathway name and corresponding P-value. Among them, HIF-1 signaling pathway has the highest P-value.

The intersection targets were analyzed using the DAVID database for Gene Ontology (GO) and KEGG pathway enrichment. GO analysis identified 3141 items, with 2726 related to biological processes (BP), primarily involving responses to organic substances, cellular responses to chemical stimuli, and oxygen-containing compounds (Fig. 6A). For cellular components (CC), 160 items were identified, predominantly related to the cytoplasm and extracellular regions (Fig. 6B). In terms of molecular functions (MF), 255 items were highlighted, mainly concerning enzyme binding, identical protein binding, and receptor binding (Fig. 6C). KEGG analysis revealed 171 enriched pathways, with key pathways including those related to cancer, lipid metabolism and atherosclerosis, and the IL-17 signaling pathway (Fig. 6D). To further elucidate the signaling pathways involving the core targets, the top 10 core targets were mapped to their associated pathways, and a "core targets-pathway" Sankey diagram was constructed (Fig. 7). Among the 171 pathways, the HIF-1 signaling pathway, TNF signaling pathway, and lipid metabolism and atherosclerosis pathways encompassed the most core targets (five targets each), with the HIF-1 signaling pathway showing the highest level of statistical significance.

### 3.5 The results of molecular docking

**Fig. 8:**
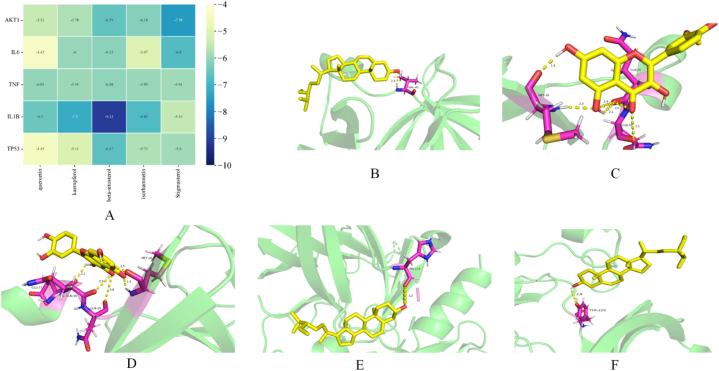
**molecular docking.** (A) Combined energy heat map; (B) IL1B and beta-sitosterol, −9.12 kcal mol^−1^; (C)IL1B and isorhamnetin, −6.28 kcal mol^−1^; (D)IL1B and kaempferol, −7.2 kcal mol^−1^; (E)TP53 and beta-sitosterol, −6.67 kcal mol^−1^; (F)AKT1 and Stigmasterol, −7.58 kcal mol^−1^.

To explore the binding interactions between the core chemical components of WJT and key target genes, molecular docking simulations were performed on the top five components and their corresponding targets (Fig. 8A). Typically, a binding energy of less than −5.0 kcal mol⁻^1^ suggests favorable binding activity between small molecule ligands and protein receptors, with lower binding energies indicating more stable docking conformations. Our results demonstrate that the binding energies for all top five chemical components and their targets were below −5.0 kcal mol⁻^1^ (Fig. 8B–F), signifying strong binding affinities between the core components and their respective targets.

### 3.6 Results of HE and IHC staining

**Fig. 9:**
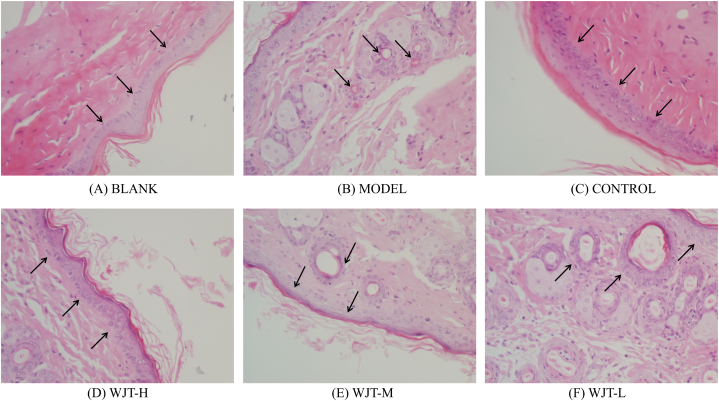
**HE staining of ectopic endometrium in each group of rats.** (A) In the blank group, ectopic endometrial cells were arranged in an orderly manner, with minimal stroma and glands, and no signs of inflammatory infiltration were observed. (B) The model group exhibited disordered arrangement of ectopic endometrial cells, with increased stromal and glandular tissue, visible neovascularization (indicated by arrows), and notable inflammatory infiltration. (C–D) The WJT-H and control groups displayed densely packed, neatly arranged ectopic endometrial cells, resembling the morphology of the blank group (indicated by arrows). (E) In the WJT-M group, there was a reduction in ectopic endometrial cells with only a small amount of inflammatory cell infiltration (indicated by arrows). (F) The WJT-L group, however, showed dispersed ectopic endometrial cells with more pronounced inflammatory infiltration, and an increase in stromal and glandular tissue (indicated by arrows), magnified at × 400.

**Fig. 10:**
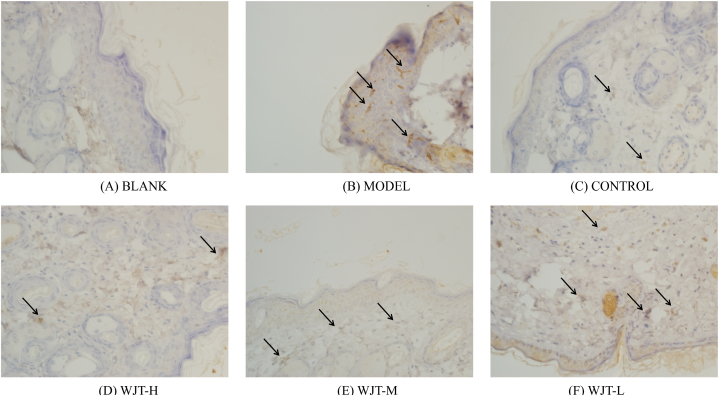
**IHC staining of HIF1A in each group of rats.** (A) In the blank group, there was negligible expression of HIF1A in the ectopic endometrium. (B) In contrast, the model group showed a marked increase in HIF1A-positive areas (indicated by the arrow). (C) The control group exhibited minimal HIF1A expression, with only a small amount of positive staining (indicated by the arrow). (D–E) In the WJT-H and WJT-M groups, the HIF1A-positive areas were significantly reduced compared to the model group (indicated by the arrow). (F) In the WJT-L group, the positive expression of HIF1A did not decrease significantly and was similar to that observed in the model group (indicated by the arrow), magnified at × 400.

**Fig. 11:**
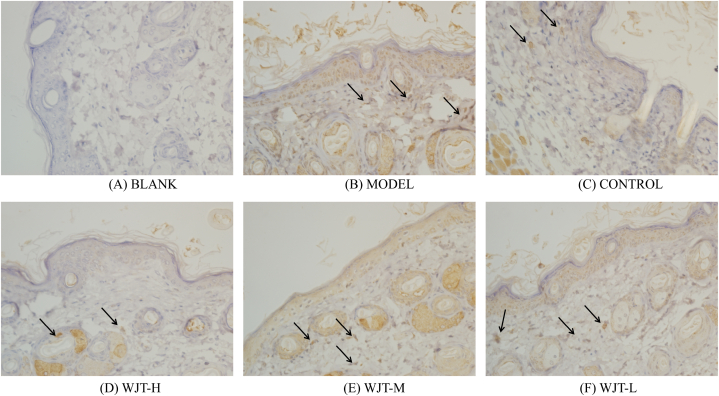
**IHC staining of STAT3 in each group of rats.** (A) The blank group showed no positive expression of STAT3 in the ectopic endometrium. (B) In contrast, the model group exhibited a significant increase in STAT3-positive areas (indicated by the arrow). (C) The control group displayed a marked reduction in STAT3-positive areas compared to the model group (indicated by the arrow). (D) In the WJT-H group, the STAT3-positive area was significantly decreased (indicated by the arrow), approaching levels observed in the blank group. (E) However, in the WJT-M group, the reduction in STAT3-positive areas was not as pronounced, with a considerable amount of positive staining still present (indicated by the arrow). (F) There was no noticeable difference in STAT3 expression between the WJT-L group and the model group (indicated by the arrow), magnified at × 400.

**Fig. 12:**
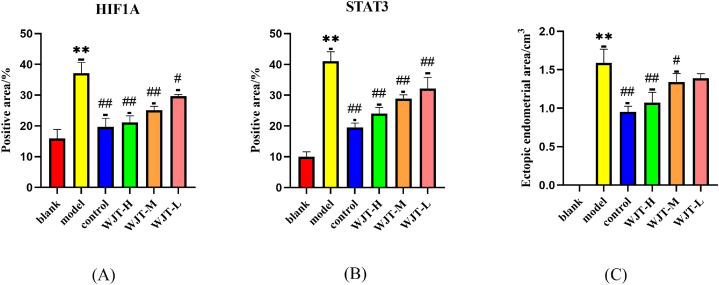
**Ectopic endometrial area and HIF1A, STAT3 positive area.** (A) The positive area of HIF1A in ectopic endometrium of rats in each group. n = 3, compared with the blank group, ∗∗*P* < 0.01; compared with the model group, ^##^*P* < 0.01, ^#^*P* < 0.05; (B) The positive area of STAT3 in ectopic endometrium of rats in each group. n = 3, compared with the blank group, ∗∗*P* < 0.01; compared with the model group, ^##^*P* < 0.01; (C) The area of ectopic endometrium in each group of rats. n = 10, compared with the blank group, ∗∗*P* < 0.01; compared with the model group, ^##^*P* < 0.01, ^#^*P* < 0.05.

Following treatment, the ectopic endometrial areas of the rats in each group were measured using a vernier caliper. The results indicated that, compared with the blank group, the ectopic endometrial area in the model group significantly increased (*P* < 0.01). In contrast, rats in the WJT-H, WJT-M, and control groups exhibited a significant reduction in ectopic endometrial area compared to the model group (*P* < 0.05), whereas no significant difference was observed between the WJT-L and model groups (Fig. 12C). Histological examination with hematoxylin and eosin (HE) staining revealed that the endometrial glandular cells in the blank group were arranged in an orderly fashion, with both glandular and stromal cells exhibiting normal growth (Fig. 9A). However, in the model and WJT-L groups, the number of endometrial glands and stromal cells was increased, with most epithelial cells displaying irregular arrangements and stromal cells densely packed (Fig. 9B, F). Notable neovascularization and inflammatory infiltrates were also observed. In contrast, the WJT-H, WJT-M, and control groups demonstrated reductions in the number of ectopic endometrial glands, stromal cells, degree of inflammatory infiltration, and overall cell morphology irregularities (Fig. 9C–E). Immunohistochemistry (IHC) staining revealed the expression patterns of HIF1A (Fig. 10) and STAT3 (Fig. 11) in the ectopic endometrial tissues of rats in each group. Compared with the blank group, the positive staining areas for HIF1A and STAT3 were significantly larger in the model group (*P* < 0.01). However, in the WJT-H, WJT-M, WJT-L, and control groups, the positive areas for HIF1A and STAT3 in the ectopic endometrial tissues were significantly reduced compared to the model group (*P* < 0.05) (Fig. 12A and B).

### 3.7 ELISA test results

**Fig. 13:**
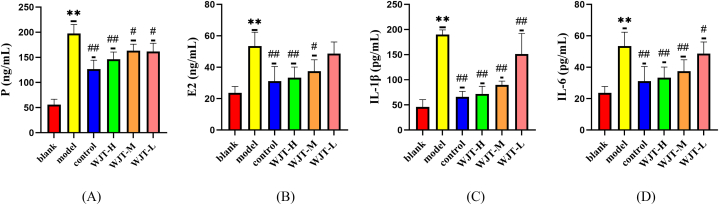
**The levels of sex hormones and inflammatory factors in each group of rats.** In the model group, levels of progesterone (P), estradiol (E2), IL-1β, and IL-6 were significantly elevated. In contrast, compared to the model group, the levels of P, E2, IL-1β, and IL-6 in the WJT-H, WJT-M, and control groups were significantly reduced. However, there was no significant difference in P levels between the WJT-L group and the model group. N = 6; ∗∗*P* < 0.01 compared to the blank group; ^##^*P* < 0.01, ^#^*P* < 0.05 compared to the model group.

EM is a hormone-dependent disorder, where abnormal fluctuations in hormone levels can trigger cytokine activation, thus promoting inflammatory responses. To investigate this, we measured the expression levels of sex hormones and inflammatory factors in the serum of rats from each group (Fig. 13A–D). In the model group, serum levels of progesterone (P) and estradiol (E2) were significantly elevated compared to the blank group (*P* < 0.01). However, in the control, WJT-H, and WJT-M groups, the levels of P and E2 were markedly reduced (P < 0.05) when compared to the model group, while no significant changes were observed in the WJT-L group (*P* > 0.05). Additionally, the levels of inflammatory markers IL-1β and IL-6 in the model group were significantly higher than those in the blank group (*P* < 0.01). In contrast, IL-1β and IL-6 levels in the control group, as well as in all WJT dose groups, were significantly decreased relative to the model group (*P* < 0.05). These findings suggest that WJT may exert its therapeutic effects by regulating both sex hormone levels and inflammatory responses.

### 3.8 Q-PCR test results

**Fig. 14:**
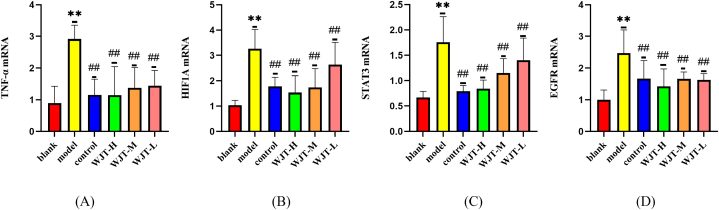
**mRNA expression levels of *TNF-α, HIF1A, STAT3* and *EGFR* in each group of rats.** In the model group, the mRNA levels of *TNF-α, HIF1A, STAT3,* and *EGFR* were significantly elevated compared to the blank group. In contrast, the mRNA levels of *TNF-α, HIF1A, STAT3,* and *EGFR* were significantly reduced in the WJT-H, WJT-M, WJT-L, and control groups compared to the model group. N = 6; ∗∗*P* < 0.01 compared to the blank group; ^##^*P* < 0.01 compared to the model group.

In order to further clarify the intervention of WJT on core targets and HIF1 pathway, we assessed the mRNA expression levels of *TNF-α, HIF1A, STAT3,* and *EGFR* in the ectopic endometrium of rats from each group using q-PCR (Fig. 14A–D). In the model group, the mRNA expression of *TNF-α, HIF1A, STAT3,* and *EGFR* was significantly upregulated compared to the blank group (P < 0.01). However, in the control, WJT-H, WJT-M, and WJT-L groups, the mRNA expression of these targets was markedly decreased compared to the model group (P < 0.01). These results suggest that WJT effectively downregulates the expression of key inflammatory and signaling molecules involved in the pathophysiology of EM.

### 3.9 Western blot test results

**Fig. 15:**
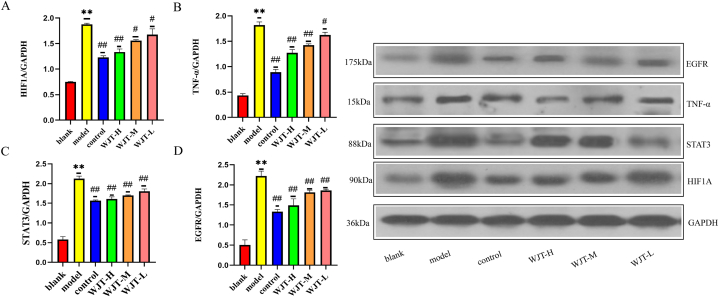
**Protein expression levels of TNF-α, HIF1A, STAT3, and EGFR in each group of rats.** The model group exhibited significantly higher protein expression of TNF-α, HIF1A, STAT3, and EGFR compared to the blank group. However, in the WJT-H, WJT-M, WJT-L, and control groups, the expression levels of these proteins were significantly reduced compared to the model group. N = 6; ∗∗*P* < 0.01 compared to the blank group; ^##^*P* < 0.01, ^#^*P* < 0.05 compared to the model group.

In addition to assessing the mRNA expression levels, we also evaluated the protein expression of TNF-α, HIF1A, STAT3, and EGFR in the ectopic endometrium of rats via Western blotting (Fig. 15A–D). In the model group, the protein expression of TNF-α, HIF1A, STAT3, and EGFR was significantly upregulated compared to the blank group (*P* < 0.01). However, in the control, WJT-H, WJT-M, and WJT-L groups, the protein expression of TNF-α, HIF1A, STAT3, and EGFR was markedly reduced compared to the model group (*P* < 0.05). These findings suggest that WJT may exert its therapeutic effects by downregulating key proteins involved in inflammation and signaling pathways associated with EM.

## 4 Discussion

EM is a prevalent and highly complex disorder, characterized by the fibrotic response of endometrial tissue bleeding, which often leads to adhesions between lesions and surrounding pelvic structures, such as the ovaries. This adhesion forms dense cysts, increasing the activity of reactive oxygen species (ROS) and cytokines, thereby exacerbating pain and contributing to the pathophysiology of the disease [20]. The resultant pain imposes significant physiological and psychological burdens on patients, severely impacting their quality of life. Currently, the primary treatment modalities for EM include ovulation suppression, hormone therapy, surgical interventions, or a combination of these approaches. However, these therapies primarily target symptom control and are not curative [21]. Wenjing Tang (WJT), a classical Chinese medicinal formula from the Han Dynasty text Synopsis of the Golden Chamber, offers a traditional therapeutic perspective. According to TCM theory, the herbs Gui Zhi, Wu Zhu Yu, and Sheng Jiang in WJT function to warm the body and promote blood circulation; Mu Dan Pi, Dang Gui, and Chuan Xiong accelerate blood flow; Mai Dong, E Jiao, and Bai Shao replenish blood and body fluids; Ren Shen and Gan Cao invigorate qi and blood, while Ban Xia is used to reduce lumps and hyperplasia. Collectively, WJT is believed to exert therapeutic effects by warming and unblocking meridians, dispelling cold, nourishing the blood, and reducing tumor-like masses. Despite these traditional explanations, the precise molecular mechanisms through which WJT exerts its effects in the treatment of EM remain unclear. Thus, this study aimed to elucidate the mechanistic pathways of WJT in treating EM through a combination of network pharmacology analysis and experimental validation. This integrative approach may provide a more comprehensive understanding of WJT's therapeutic potential in managing EM.

A total of 10 core chemical components have been identified through network pharmacology analysis. Notably, in TCM theory, EM is thought to arise from a deficiency of yang qi. In WJT, Gui Zhi and Wu Zhu Yu are the primary herbs used to replenish yang qi, and they are present in significant quantities in the formula. The main chemical constituents of these two herbs include quercetin, sitosterol, beta-sitosterol, isorhamnetin, and rutaecarpine—compounds that are likely key to WJT's therapeutic effects in treating EM. Beta-sitosterol, stigmasterol, and sitosterol are cholesterol-like substances commonly found in various natural environments. These compounds possess a wide range of pharmacological activities, including anti-inflammatory, antioxidant, cholesterol-lowering, blood glucose-lowering, and immune-regulatory effects [[22], [23], [24]]. Previous studies have demonstrated that beta-sitosterol can induce apoptosis in ectopic endometrial stromal cells (hEM15A), inhibit cell proliferation, and upregulate the expression of Smad7 [25]. Flavonoid compounds such as quercetin, kaempferol, isorhamnetin, and glabrene are known for their anti-inflammatory and antioxidant properties, as well as their ability to improve glucose and lipid metabolism [26]. Research has shown that quercetin can significantly reduce levels of 17β-E2, ERα, ERβ, PR, FSH, and LH in EM model rats [[27], [28], [29]]. Rutaecarpine, classified as an alkaloid, has also been shown to inhibit oxidative stress and inflammation [[30], [31], [32]]. These findings suggest that the therapeutic effects of WJT in treating EM may be closely linked to its ability to inhibit inflammation and oxidative stress, providing a molecular basis for its use in TCM.

A total of 10 core targets were identified through PPI analysis, and subsequent enrichment analysis revealed that these core targets are closely related to the HIF-1 signaling pathway. HIF-1 is a critical regulatory factor in the hypoxic-ischemic response, consisting of two subunits: the oxygen-sensitive HIF-1α and the oxygen-insensitive HIF-1β [33]. Under normoxic conditions, HIF-1α undergoes hydroxylation at proline residues 402 and 564, which triggers its degradation via the ubiquitin-proteasome pathway [34]. However, under hypoxic conditions, HIF-1α evades oxygen-dependent hydroxylation and proteasomal degradation, leading to its accumulation in the cytoplasm. It then translocates to the nucleus, where it binds with HIF-1β to form a transcription factor complex. This complex promotes the expression of genes such as EGFR, EPO, and glycolytic enzymes, enhancing endothelial cell proliferation and migration, which contributes to endometrial growth and the progression of EM [[35], [36], [37]]. Moreover, EM is an estrogen-dependent inflammatory disorder associated with elevated levels of pro-inflammatory cytokines, such as IL-6, IL-1β, and TNF-α, in both the affected tissues and peritoneal fluid [38]. Studies have shown that IL-6 levels are significantly higher in the endometrial stromal cells of patients with EM compared to those without the disease [39].

STAT3, a key player in cytokine signaling, is located on chromosome 17 and encodes a protein that belongs to the STAT family. STAT3 is activated by the phosphorylation of various cytokines and growth factors, including IFN, EGF, IL-6, HGF, and BMP2 [40,41]. Both IL-6 and EGFR can activate signaling pathways mediated by STAT3, which, once activated, translocates to the nucleus. There, it forms homo- or heterodimers that bind to promoter regions of target genes like HIF1A, promoting processes such as stem cell proliferation, angiogenesis, and immune regulation—factors that contribute to the pathogenesis of EM [42,43]. Furthermore, research has demonstrated that activated STAT3 can inhibit the maturation of dendritic cells, a process mediated by the anti-inflammatory cytokine IL-37 in EM. Inhibiting STAT3-mediated HIF1A expression has been shown to alleviate the inflammatory response associated with EM [44]. Our findings suggest that WJT can significantly reduce the expression of TNF-α, HIF1A, STAT3, and EGFR at both the mRNA and protein levels. This indicates that WJT may exert its therapeutic effects on EM by inhibiting the HIF-1 signaling pathway and reducing the associated inflammatory response.

## 5 Conclusion

In conclusion, this study identified 10 core chemical components, including quercetin, kaempferol, and beta-sitosterol, along with 10 key targets such as AKT1, HIF1A, and STAT3, through network pharmacology analysis. These core targets were found to be significantly enriched in the HIF-1 signaling pathway. WJT demonstrated the potential to modulate sex hormone levels, inhibit the activation of the HIF-1 pathway, and suppress the expression of inflammatory factors. These findings suggest that WJT may have a promising role in the prevention and treatment of EM by targeting multiple pathways associated with the disease's pathogenesis.

## CRediT authorship contribution statement

**Xufang Hu:** Writing – original draft. **Xiaoya Guo:** Conceptualization. **Dongxu Wei:** Investigation. **Jingyi Yue:** Methodology. **Jian Zhang:** Writing – original draft. **Bing Wang:** Writing – review & editing.

## Ethics statement

This study was approved by the Experimental Ethics Committee of Heilongjiang University of Traditional Chinese Medicine (Approval No: SYXK2020-004).

## Conflict of interest statement

The authors declare that there are no conflicts of interest. Data availability statement The original contributions presented in the study are included in the article, further inquiries can be directed to the corresponding author.

## Data availability statement

All data generated or analyzed during this study are available from the corresponding author on reasonable request.

## Funding

This study is funded by the fifth batch of national clinical excellent talents training program for traditional Chinese medicine by the China administration of traditional Chinese medicine ([2022] No. 239); research project of Heilongjiang provincial administration of traditional Chinese medicine (ZYW2022-048); Heilongjiang province higher education teaching reform research project (SJGY20210827).

## Declaration of competing interest

The authors declare that they have no known competing financial interests or personal relationships that could have appeared to influence the work reported in this paper.
