# Supplementary figures and images for: The mechanism of wen jing tang in the treatment of endometriosis: Insights from network pharmacology and experimental validation
Source: Heliyon. 2024 Oct 17;10(21):e39292. doi: 10.1016/j.heliyon.2024.e39292 (PMC11546154; doi:10.1016/j.heliyon.2024.e39292)

TNF- $\alpha$

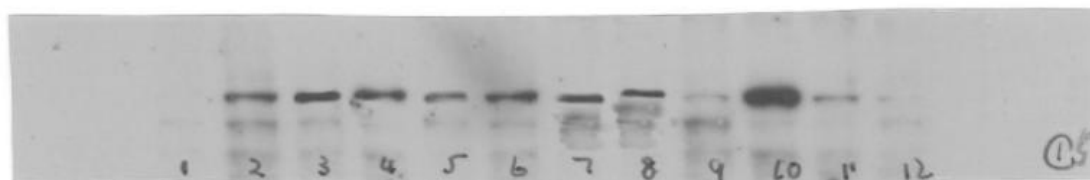

STAT3

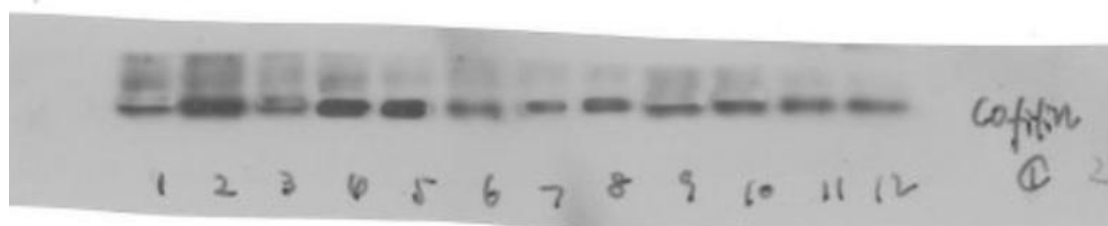

HIF1A

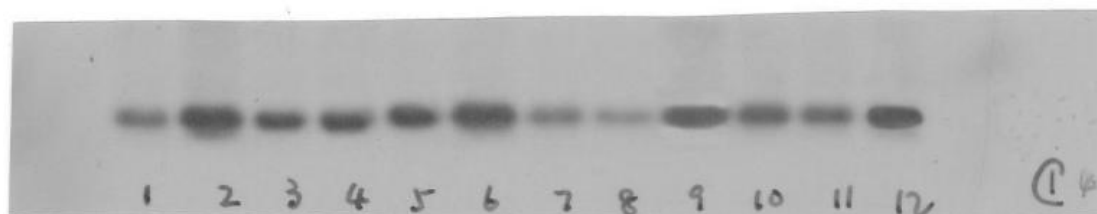

GAPDH

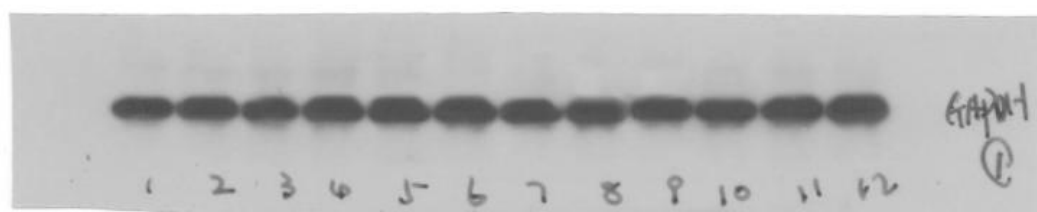

EGFR

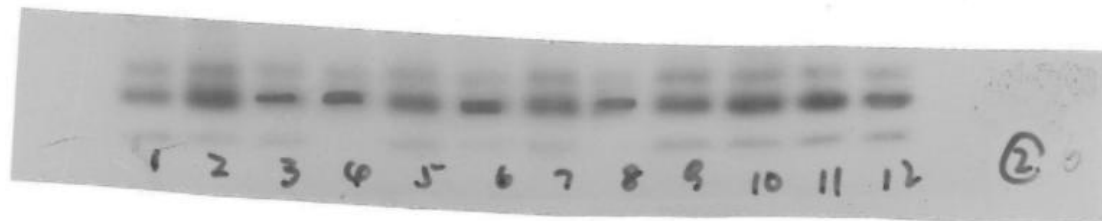

TNF- $\alpha$

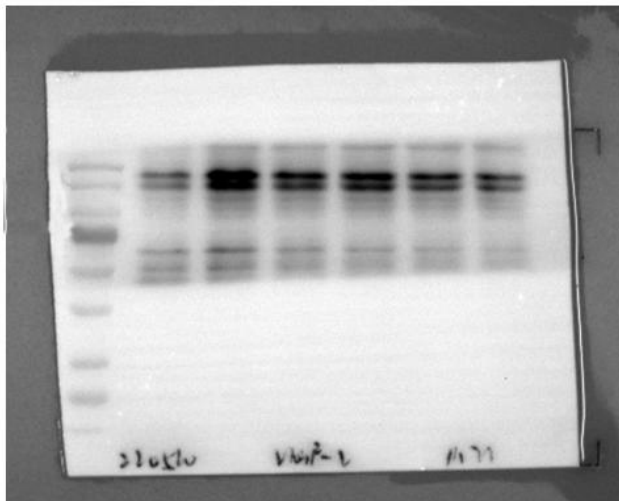

STAT3

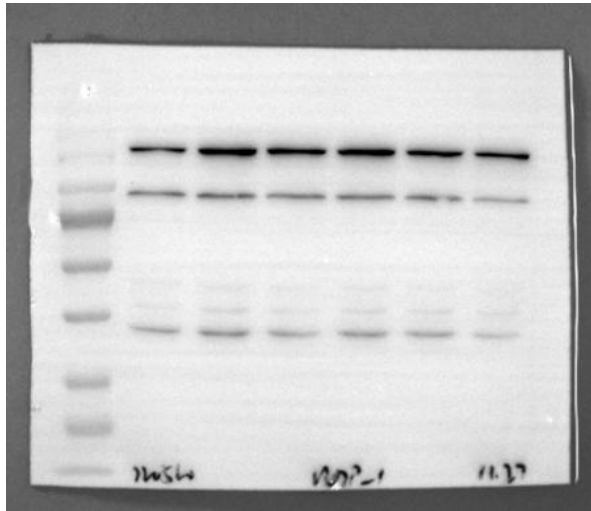

HIF1A

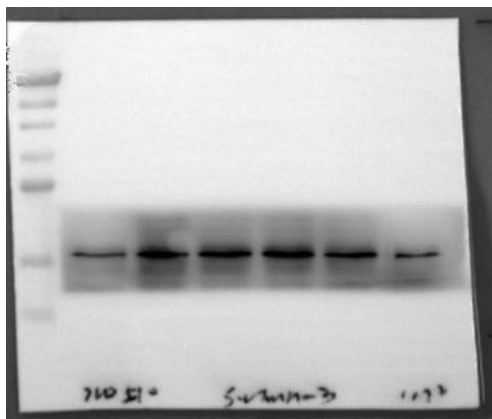

EGFR

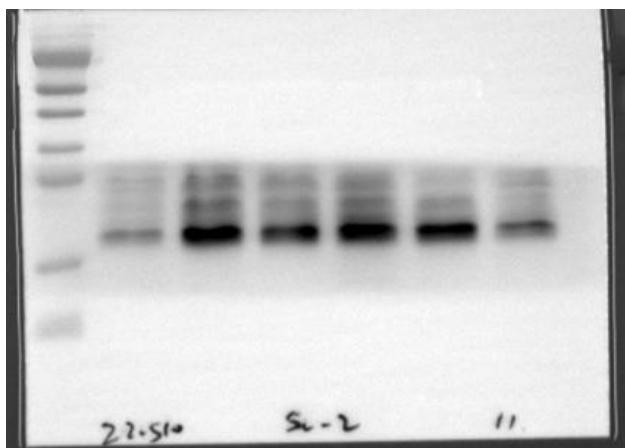

GAPDH

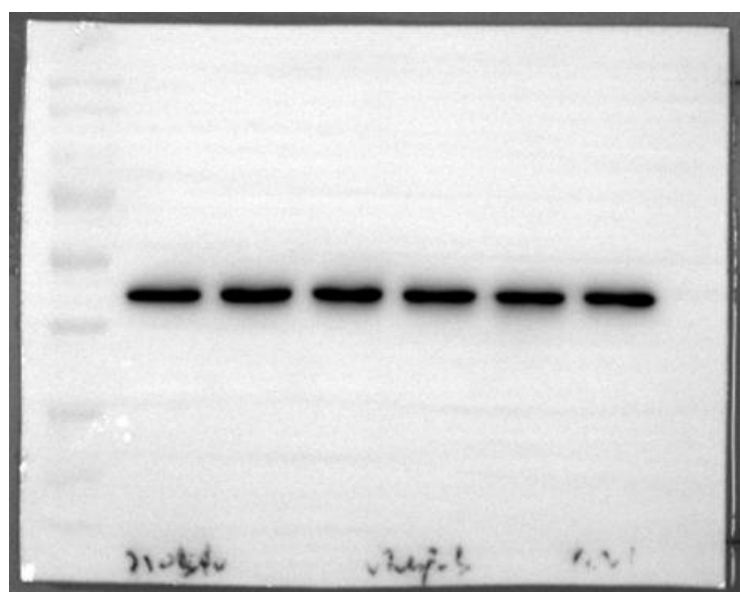

Supplement: Multimedia component 1 [file mmc1.pdf]
